# Supplementary figures and images for: Restriction of HIV-1 infectivity by interferon and IFITM3 is counteracted by Nef
Source: bioRxiv. 2025 May 15:2025.05.15.654345. Preprint. [Version 1] doi: 10.1101/2025.05.15.654345 (PMC12132579; doi:10.1101/2025.05.15.654345)

Supplemental Figure 1

A

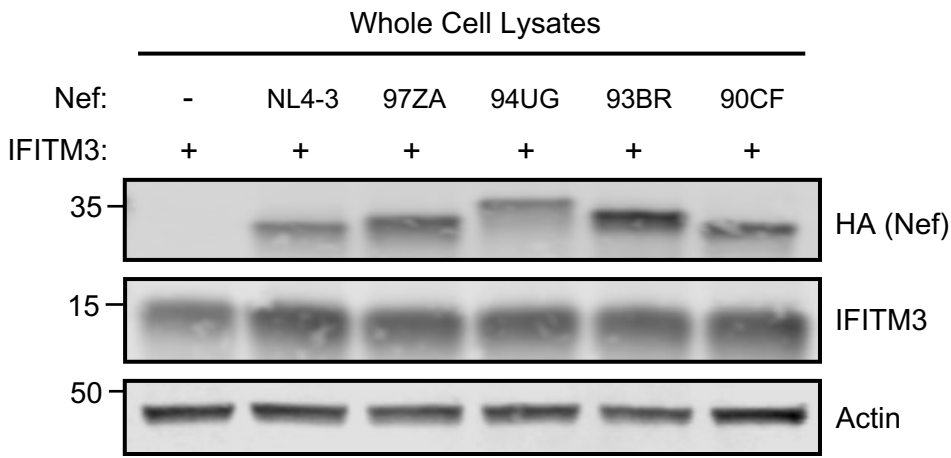

B

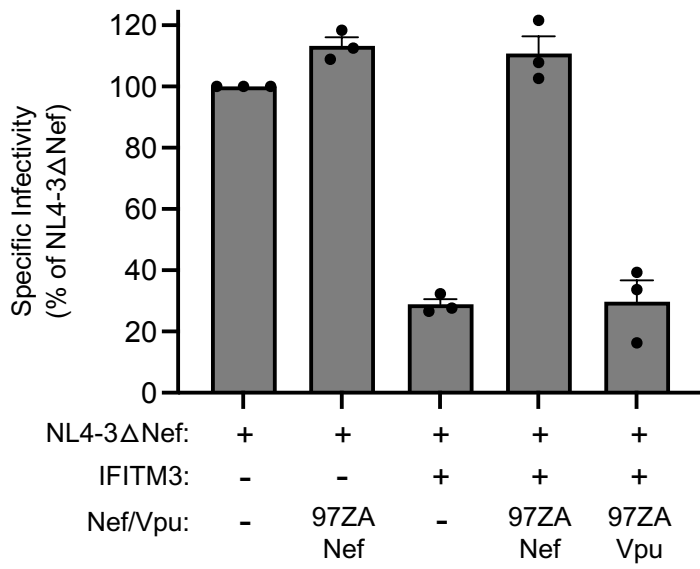

C

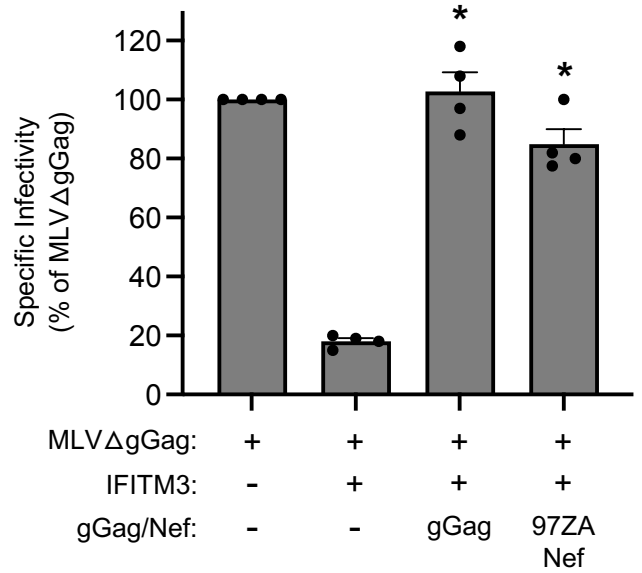

D

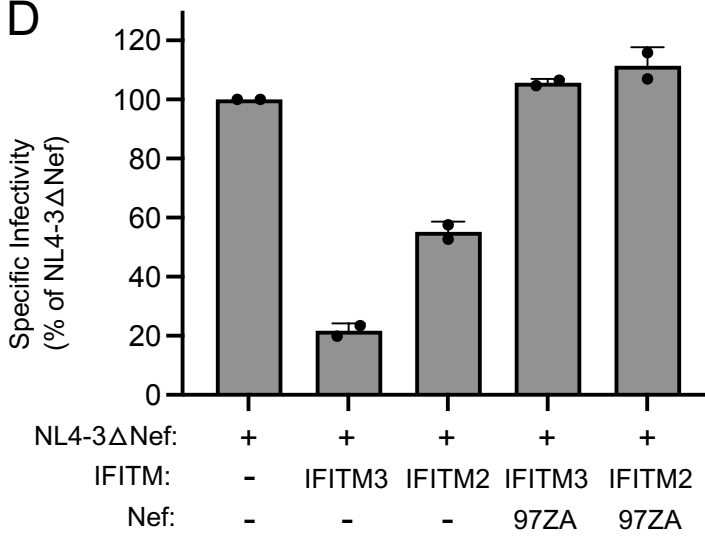

E

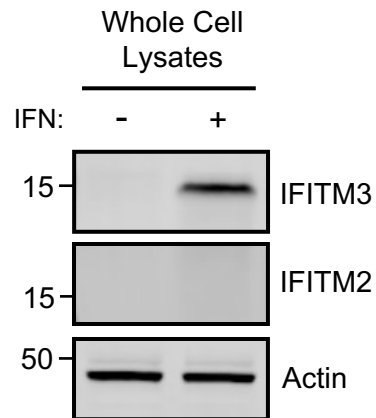

F

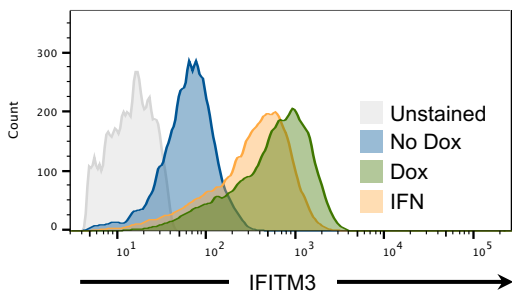

Supplement: Supplement 1 — Supplemental Figure 1: (A) HEK293T cells were co-transfected with pCMV-IFITM3 (0.50 μg) and pBJ-Nef-HA encoding the indicate Nef protein (0.25 μg), and whole cell lysates were subjected to SDS-PAGE and immunoblotting with anti-HA, anti-IFITM3, and anti-Actin (which served as loading control). Numbers and tick marks left of blots indicate position and size (in kilodaltons) of protein standard in ladder. (B) HEK293T cells were co-transfected with NL4–3△Nef (2.0 μg), pCMV-IFITM3 or Empty Vector (0.5 μg), and pBJ-97ZA Nef-HA or pBJ-97ZA Vpu-HA (0.25 μg). Produced virus-containing supernatants were harvested 24 hours post-transfection and quantified by viral p24 ELISA. 25 ng p24 equivalent of fresh virus-containing supernatants were added to TZM-bl cells, and infection was scored by anti-Gag immunostaining at 48 hours post-inoculation. Virus infectivity of each condition is shown as mean and standard deviation (normalized relative to NL4–3△Nef alone, which was set to 100%). Filled circles represent biological replicates (independent transfections). (C) HEK293T cells were co-transfected with MLV△glycoGag (2.5 μg), pBabeLuc (0.6 μg), pCMV-Xenogp85 (xenotropic Env) (0.5 μg), and pCMV-IFITM3 or Empty Vector (0.5 μg), and where indicated, pCMV-glycoGag-Myc (0.25 μg) or pBJ-97ZA Nef-HA (0.25 μg). Produced virus-containing supernatants were harvested 48 hours post-transfection and quantified by viral Gag immunoblotting with anti-p30 of pelleted viruses. Equal volumes of p24 equivalent of fresh virus-containing supernatants were added to HT1080-mCAT1cells, and infection was scored by luciferase assay at 48 hours post-inoculation. Luciferase values were divided by Gag immunoblot intensity to derive a specific infectivity measurement. Virus infectivity of each condition is shown as mean and standard deviation (normalized relative to MLV△glycoGag alone, which was set to 100%). Differences that were statistically different from the indicated condition and MLV△glycoGag + IFITM3 are i [file media-1.pdf]

Supplemental Figure 2

A

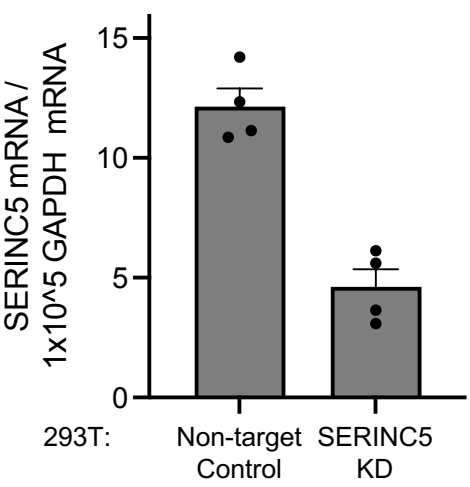

B

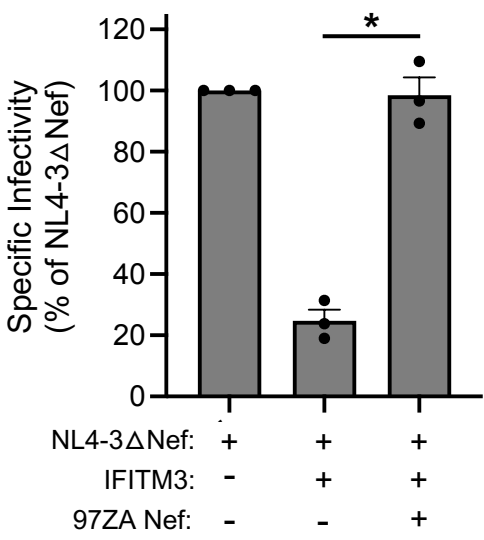

Supplement: Supplement 2 — Supplemental Figure 2: (A) Stable SERINC5 knockdown was performed in HEK293T cells as previously described, and knockdown was assessed by quantitative RT-PCR (70). SERINC5 mRNA levels per 1×10^5 copies of GAPDH mRNA were plotted. (B) SERINC5 knockdown HEK293T cells were co-transfected with NL4–3△Nef (2.0 μg), pCMV-IFITM3 or Empty Vector (0.5 μg), and pBJ-97ZA Nef-HA (0.25 μg). Produced virus-containing supernatants were harvested 24 hours post-transfection and quantified by viral p24 ELISA. 25 ng p24 equivalent of fresh virus-containing supernatants were added to TZM-bl cells, and infection was scored by anti-Gag immunostaining at 48 hours post-inoculation. Virus infectivity of each condition is shown as mean and standard deviation (normalized relative to NL4–3△Nef alone, which was set to 100%). Differences between the indicated conditions that were statistically significant as measured by student’s T test are indicated by (*) (p < 0.05). Filled circles represent biological replicates (independent transfections). [file media-2.pdf]

Supplemental Figure 3

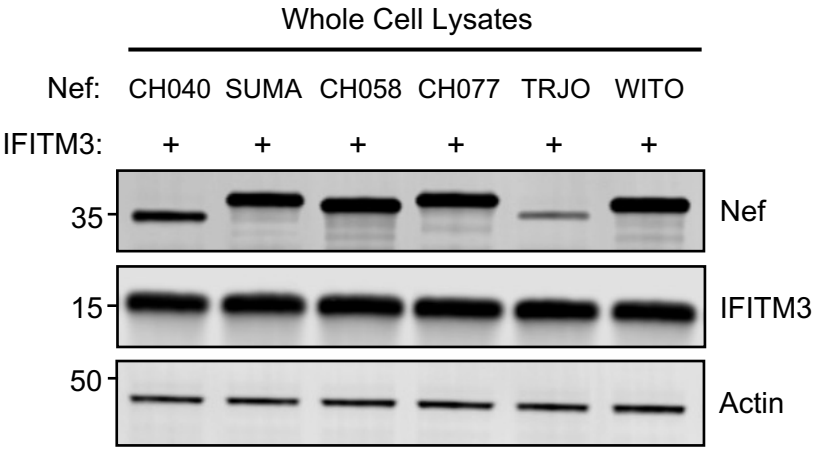

Supplement: Supplement 3 — Supplemental Figure 3: HEK293T cells were co-transfected with pCMV-IFITM3 (0.50 μg) and pCl-Nef encoding the indicate Nef protein (0.25 μg), and whole cell lysates were subjected to SDS-PAGE and immunoblotting with anti-Nef, anti-IFITM3, and anti-Actin (which served as loading control). Numbers and tick marks left of blots indicate position and size (in kilodaltons) of protein standard in ladder. [file media-3.pdf]
